# The Asthma Risk Gene, *GSDMB*, Promotes Mitochondrial DNA-induced *ISGs* Expression

**DOI:** 10.35534/jrbtm.2024.10005

**Published:** 2024-03-31

**Authors:** Tao Liu, Julian Hecker, Siqi Liu, Xianliang Rui, Nathan Boyer, Jennifer Wang, Yuzhen Yu, Yihan Zhang, Hongmei Mou, Luis Guillermo Gomez-Escobar, Augustine M.K. Choi, Benjamin A. Raby, Scott T. Weiss, Xiaobo Zhou

**Affiliations:** 1 Channing Division of Network Medicine, Brigham and Women’s Hospital and Harvard Medical School, Boston, MA 02115, USA; 2 The Mucosal Immunology and Biology Research Center, Massachusetts General Hospital and Harvard Medical School, Boston, MA 02115, USA; 3 Weil Cornell Medical School, Joan and Sanford I. Weill Department of Medicine, New York, NY 10065, USA; 4 Division of Pulmonary Medicine, Department of Pediatrics, Boston Children’s Hospital and Harvard Medical School, Boston, MA 02115, USA

**Keywords:** cGAS-STING pathway, GSDMB, ISGs, Asthma, Airway inflammation

## Abstract

Released mitochondrial DNA (mtDNA) in cells activates cGAS-STING pathway, which induces expression of interferon-stimulated genes (ISGs) and thereby promotes inflammation, as frequently seen in asthmatic airways. However, whether the genetic determinant, Gasdermin B (GSDMB), the most replicated asthma risk gene, regulates this pathway remains unknown. We set out to determine whether and how GSDMB regulates mtDNA-activated cGAS-STING pathway and subsequent *ISGs* induction in human airway epithelial cells. Using qPCR, ELISA, native polyacrylamide gel electrophoresis, co-immunoprecipitation and immunofluorescence assays, we evaluated the regulation of GSDMB on cGAS-STING pathway in both BEAS-2B cells and primary normal human bronchial epithelial cells (nHBEs). mtDNA was extracted in plasma samples from human asthmatics and the correlation between mtDNA levels and eosinophil counts was analyzed. *GSDMB* is significantly associated with *RANTES* expression in asthmatic nasal epithelial brushing samples from the Genes-environments and Admixture in Latino Americans (GALA) II study. Over-expression of *GSDMB* promotes DNA-induced IFN and *ISGs expression* in bronchial epithelial BEAS-2B cells and nHBEs. Conversely, knockout of *GSDMB* led to weakened induction of *interferon* (IFNs) and *ISGs* in BEAS-2B cells. Mechanistically, GSDMB interacts with the C-terminus of STING, promoting the translocation of STING to Golgi, leading to the phosphorylation of IRF3 and induction of *IFNs* and *ISGs*. mtDNA copy number in serum from asthmatics was significantly correlated with blood eosinophil counts especially in male subjects. GSDMB promotes the activation of mtDNA and poly (dA:dT)-induced activation of cGAS-STING pathway in airway epithelial cells, leading to enhanced induction of *ISGs.*

## 1. Introduction

Asthma, the most prevalent airway disease in school-age children, imposes a significant economic burden globally [1]. Despite effective treatment to manage asthma symptoms, the disease persists with no cure. Genetic predisposition determines the risk for individuals to develop asthma with single nucleotide polymorphisms (SNPs) at chromosome 17q21 as the most reproducible and significant asthma susceptibility locus associated with early-onset childhood asthma [2]. Risk alleles in these 17q21 SNPs are associated with asthma severity and exacerbation and increased expression of the gasdermin B (GSDMB) gene in airway epithelial cells [3–6]. Pyroptosis is a form of inflammatory cell death characterized by Gasdermins (GSDMs)-mediated cell lysis and involves the activation of inflammatory caspases [7,8]. GSDMB, unique to humans without mouse homolog, belongs to Gasdermins (GSDMs) family, which induces pyroptosis in tumors or upon bacterial infections [9–12]. Non-pyroptosis-related function of GSDMB was reported in inflammatory bowel disease (IBD), through regulation of restoration of the intestinal lining [13,14]. Interestingly, over expression of human *GSDMB* isoform 1 lacking exon 6 in mice led to airway remodeling without profound inflammation [13]. However, whether and how full length GSDMB promotes airway inflammation in patients with asthma remains unclear.

Proper activation of cGAS-STING pathway is a vital mechanism responds to aberrant cytosolic DNA, which are majorly originated from DNA virus or dysregulated self-DNA. However, over-activated cGAS-STING pathway promotes inflammation that accelerates a variety of inflammatory diseases, such as amyotrophic lateral sclerosis [15], doxorubicin-induced cardiotoxicity (DIC) [16], or systemic lupus erythematosus [17]. Mechanically, cGAS directly binds to DNA, converting guanosine 5´-triphosphate (GTP) and adenosine 5´-triphosphate (ATP) into the second messenger 2’ 3’ cyclic GMP-AMP (cGAMP), which binds to and activates stimulator of interferon genes (STING) [18]. Subsequently, STING oligomerizes and translocates from the endoplasmic reticulum (ER) to the Golgi apparatus where it recruits TBK1, phosphorylating IRF3 [19–26], leading to the translocation of phosphorylated and dimerized IRF3 into nucleus activating the transcription of type I and type III *IFNs*, inducing expression of *ISGs* [27–30]. The levels of *ISGs* expression, are elevated in asthma and associated with reduced lung function and ER stress [31,32]. For example, *RANTES* (also known as *CCL5*), an important pro-inflammatory *ISG* with increased expression in asthmatics, contributes to the recruitment of eosinophils in allergic airway inflammation [33–36]. Also, *RANTES* contribute to neutrophilic inflammation in asthma, potentially serving as a bridge between type 1 and type 2 inflammation [37]. Moreover, targeting RANTES-responsive cells within the lung prevents chronic fungal asthma [38]. However, how *ISGs* is dysregulated in asthma remain unclear.

In various pulmonary diseases, including coronavirus disease 2019 (COVID-19) [39], idiopathic pulmonary fibrosis (IPF) [40,41], extrapulmonary sarcoidosis [42], as well as asthma [43–46], aberrant release of mitochondrial DNA (mtDNA) through the BAK/BAX macropores was reported, which is a potent agonist of cGAS-STING pathway, potentiating inflammation in response to stress or injury [47–52]. While the genetic analyses have identified the association between GSDMB and interferon response [53], the molecular mechanisms and ensuing biological effects of GSDMB in mtDNA-induced cGAS-STING pathway in the context of asthma remain largely undefined.

In this study, we found that GSDMB promotes mtDNA-induced IFN production and subsequent *ISGs* activation through regulating the cGAS-STING pathway in human bronchial epithelial cells. Mechanically, GSDMB interacts with STING, facilitating its Golgi localization and subsequent TBK1 recruitment, without affecting cGAMP binding or STING oligomerization. Hence, our findings provide novel molecular insights into how the genetic determinant GSDMB regulates lung inflammation in asthma development, suggesting a promising therapeutic strategy for treating airway inflammation in genetically susceptible asthmatics.

## 2. Materials and Methods

### 2.1. Cells

HEK293, HEK293FT and BEAS-2B cells were grown in DMEM (Life Technologies; Carlsbad, CA, USA) with 10% (*v*/*v*) fetal bovine serum (Thermo Fisher;Pittsburgh, PA, USA), 1% Penicillin-Streptomycin-Glutamine (Thermo Fisher). As previously outlined [54,55], normal human bronchial epithelial cells (nHBEs) were cultured in Small Airway Epithelial Cell Medium (Promocell; Heidelberg, Germany) with the addition of 1.0 μM A8301(Tocris Bioscience; Bristol, UK), 0.5 μM CHIR99021(Sigma-Aldrich, St Louis, MO, USA) and 5 μM Y27632 (Sigma-Aldrich) on plates pre-coated with laminin-enriched 804G-conditioned medium.

### 2.2. Establishment of the stable cell line with overexpression of GSDMB

Stable cell lines with *GSDMB*-overexpression were established by infecting BEAS-2B cells or nHBEs with Gasdermin like (*GSDMB*) Human Tagged ORF Clone Lentiviral Particle (Cat. RC219044L1V, Origene; Rockville, MD, USA). The expression of GSDMB in the cells was measured by Western blot analysis or qPCR. All details for antibodies are listed in Table S1.

### 2.3. Generation of Mitochondrial DNA Deficient (ρ0) Cells

To deplete mtDNA from cells, BEAS-2B cells were treated with EtBr (100 ng/mL, Sigma-Aldrich) in medium supplemented with pyruvate (100 μg/mL, Sigma-Aldrich) and uridine (50 μg/mL, Sigma-Aldrich) for 7 days at 37 °C with 5% CO_2_ in an incubator. The total DNA of indicated cells was obtained by using QIAamp DNA Micro Kit (QIAGEN; Venlo, The Netherlands) per the manufacturer’s instructions. The depletion of mitochondrial DNA was quantified by RT-PCR with mtDNA (D-LOOP) primer as previously described [43] (Table S2).

### 2.4. mtDNA Extraction from Cultured Cells

Mitochondria were extracted from BEAS-2B cells or HEK 293 cells using the mitochondrial isolation kit (Abcam, # ab110170; Waltham, MA, USA) based on manufacture’s protocol. The freshly isolated mitochondria were then purified with the QIAamp DSP DNA Blood Mini Kit (Qiagen, Cat. No. 61104) according to the manufacturer’s instructions.

### 2.5. Gene Expression Correlation Analysis in Nasal Epithelial Cells

RNA-sequencing data in nasal epithelium was obtained from the Genes-environments & Admixture in Latino Americans (GALA) II study (*n* = 695, GSE152004) [56]. This dataset contains normalized expression data for 441 asthmatics and 254 healthy controls. We extracted the expression data for the 12 genes including *RANTES*, *CCL8*, *CXCL9*, *CXCL10*, *ISG15*, *ISG20*, *GBP1*, *GBP2*, *IRF1*, *IRF7*, *IFNγ* and *IFNλ2.* We then computed the log2 transformed values of gene expression for correlation analysis between *GSDMB* and other genes based on the log2 transformed values. To determine whether co-expression differs between cases and controls, we applied a permutation-based approach where the observed absolute difference in co-expressions between cases and controls (measured as the Pearson correlation of the log2 transformed data) is compared to the corresponding absolute difference after shuffling the case/control status randomly. Based on 10,000 permutations, we computed empirical *p*-values for this comparison.

### 2.6. Correlation Analysis between mtDNA and Various Clinical Phenotypes in Asthmatics

mtDNA was extracted and quantified from plasma samples from asthmatic subjects enrolled in the Costa Rican Asthma cohort. The study of this cohort was approved by the Institutional Review Boards of the Brigham and Women’s Hospital (Boston, MA) entitled “the Genetic Epidemiology of Asthma in Costa Rica”: Protocol #:2000P001130. To analyze the correlation of mtDNA copy number with various clinical characteristic in asthma patients (*n* = 375), we first filtered out samples with extremely high mitochondrial DNA (mtDNA) measurements (> 10,000 copies/μL plasma). For final analysis, 357 samples were kept for the association analysis between mtDNA levels and various asthma-related outcomes. We also performed an association analysis between mtDNA and eosinophil counts in asthmatics. Both analyses adjusted for age, sex, and height.

## 3. Results

### 3.1. GSDMB Elicits dsDNA-induced ISGs Expression in Human Airway Epithelium

**Figure 1.**
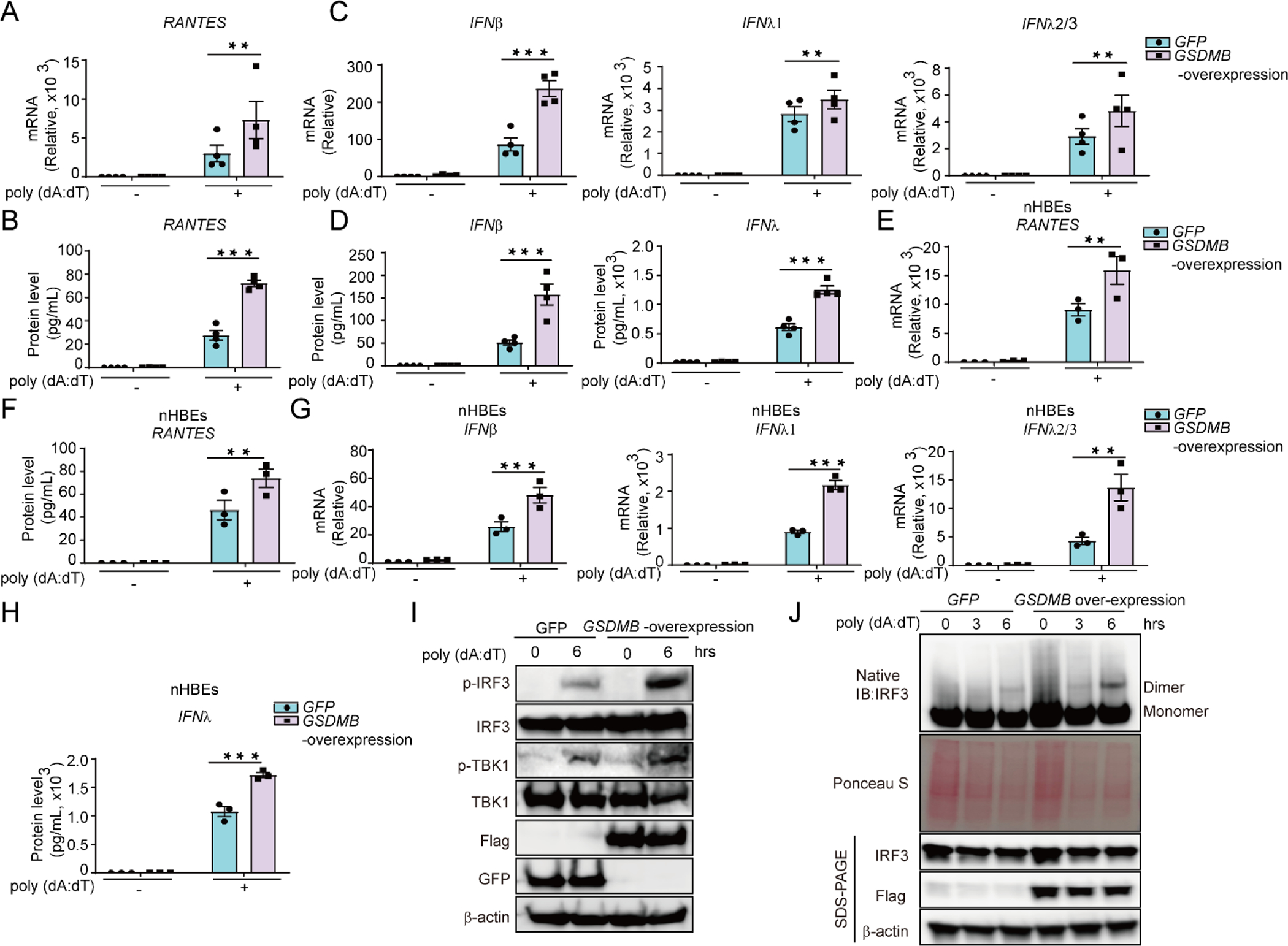
GSDMB enhances the induction of IFNs and ISGs by dsDNA. (**A**,**B**) The mRNA (**A**) or protein levels of RANTES (**B**) were evaluated in BEAS-2B cells transfected with poly (dA:dT) for 6 hours (h). Both GFP-transfected control cells and stable *GSDMB*-overexpressing cells were used. (**C**,**D**) Expression of *IFNs*, including *IFNβ*, *IFNλ1*, *IFNλ2/3* (**C**) and protein levels of IFNβ, IFNλ1/3 (**D**) were measured in BEAS-2B cells with overexpression of *GSDMB* and transfection of poly (dA:dT) for 6 h. (**E**,**F**) The mRNA (**E**) or protein levels of RANTES (**F**) were assessed in nHBEs with stable overexpression of *GSDMB* post transfection of poly (dA:dT) for 6 h. (**G**,**H**) Expression of *IFNs*, including *IFNβ*, *IFNλ1*, *IFNλ2/3* (**G**) and protein levels of IFNλ 1/3 (**H**) were assessed in nHBEs transfected with poly (dA:dT) for 6 h with or without stable overexpression of *GSDMB*. (**I**) The levels of IRF3 and TBK1 phosphorylation were determined by immunoblotting in GFP- or *GSDMB*-overexpressing BEAS-2B cells post-transfection with poly (dA:dT) for 6 h. (**J**) IRF3 dimerization was examined in BEAS-2B cells overexpressing *GSDMB* and transfected with poly (dA:dT) for 0, 3, and 6 hours (h) by resolving protein extracts using native PAGE analysis. Cells expressing GFP were used as a control. Data in A-H is presented as means ± SEM from four independent biological replicates. *p < 0.05; **p < 0.01 (two-way ANOVA). The immunoblot data shown are representatives from two independent biological experiments.

Increased *ISGs* expression and hyper-inflammation in airways are closely linked to the decline of lung function in asthma [32,57]. To determine whether GSDMB regulates *ISGs* expression, we engineered a *GSDMB*-over-expressing bronchial epithelial BEAS-2B cell line. As compared to the control group, upon transfection of poly (dA:dT), *GSDMB*-over-expressing cells showed exuberant induction of *ISGs* including *RANTES* and 2’–5’-Oligoadenylate Synthetase Like (OASL) (Figures 1A,B and S1A). Moreover, expression of RANTES was more significantly correlated with *GSDMB* in asthmatic nasal epithelial cells compared to control subjects in the Genes-environments and Admixture in Latino Americans (GALA) II study (*n* = 254 controls and 441 with asthma) [56,58] (Table S3). In addition to *ISGs*, overexpression of *GSDMB* also resulted in the remarkable induction of IFNs (*IFNβ*, *IFNλ1* and *IFNλ2/3*) (Figure 1C,D). Furthermore, overexpression of *GSDMB* (Figure S1B) promotes induction of *RANTES* and *IFNs* in nHBEs (Figure 1E,F and 1G,H). Consistently, the phosphorylated TBK1/IRF3 and subsequent dimerization of IRF3, important markers to indicate IFN response [59], are significantly upregulated in *GSDMB*-overexpressing cells (Figure 1I,J), suggesting that GSDMB promotes induction of *IFNs* and *ISGs* by poly (dA:dT) through activating the cGAS-STING pathway.

### 3.2. GSDMB Promotes cGAS-STING Signaling Independent of Pyroptosis, without Affecting ER Stress

Given that GSDMB has been previously documented to facilitate inflammasome activation [11,60–63], leading to pyroptosis and may consequently amplify subsequent *ISGs* expression [62,64], we thus evaluated whether the regulation of the cGAS/STING pathway by GSDMB depends on pyroptosis. Surprisingly, no evidence showed that GSDMB promotes poly (dA:dT) induced pyroptosis, indicated by intake of propidium iodide (PI) (Figure S2A), rupture of the cell membrane (Figure S2A), release of lactate dehydrogenase (LDH) (Figure S2B) and secretion of interleukin-1β (IL-1β) (Figure S2C). In line with this, the caspase-1 inhibitor YVAD failed to inhibit the induction of *RANTES* by poly (dA:dT) (Figure S2D). This observation indicates that inflammasome activation is likely dispensable for GSDMB’s regulation on poly (dA:dT)-induced activation of cGAS/STING pathway in BEAS-2B cells.

Endoplasmic reticulum (ER) stress is a hall-marker of inflammation and exacerbates tissue damage in a wide spectrum of human diseases [65]. In asthma, particularly among patients with mild asthma, ER stress is associated with both type 2 inflammation and ISG expression [32]. As ER stress could be triggered by an overexpression system [66], which can further regulate the interferon (IFN) response, we conducted an assessment of ER stress in the presence or absence of GSDMB. However, comparable expression levels of the ER stress marker, splicing of X-Box binding protein 1 (XBP1) observed in both control and *GSDMB*-overexpressing cells, indicating no effects of GSDMB on the activation of ER stress (Figure S3).

### 3.3. Deficiency of GSDMB Suppresses IFN Response and ISGs Activation

**Figure 2.**
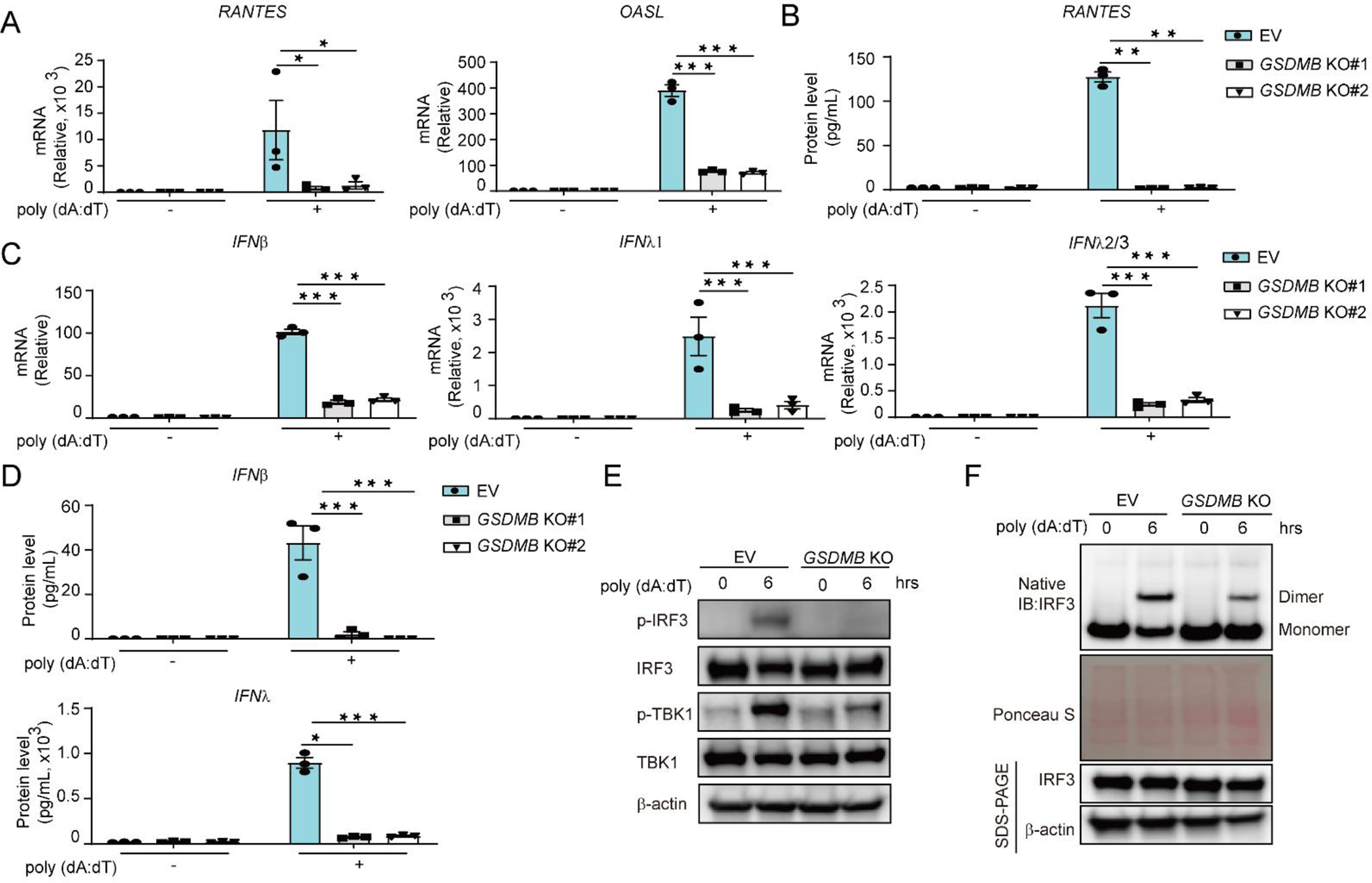
Deficiency of *GSDMB* attenuates dsDNA-induced *ISGs* expression. (**A**,**B**) Expression of *RANTES* and *OASL* (**A**) or protein levels of RANTES (**B**) were measured in empty vector (EV) or *GSDMB* knockout (KO) BEAS-2B cells transfected with poly (dA:dT) for 6 hours (h). (**C**,**D**) BEAS-2B cells with or without GSDMB expression were transfected with poly (dA:dT) for 6 h, and mRNA levels of *IFNβ*, *IFNλ1* and *IFNλ2/3* (**C**) or IFNβ and IFNλ protein levels (**D**) were measured by RT-PCR or ELISA respectively. (**E**) The phosphorylation of IRF3 and TBK1 was detected in empty vector (EV)- or *GSDMB* KO cells transfected with poly (dA:dT) for 6 h. (**F**) The IRF3 dimerization was examined in BEAS-2B cells with or without GSDMB expression after transfection with poly (dA:dT) at indicated time point. Cells expressing empty vector (EV) were used as a control. Means ± SEM shown in A-D were from three independent biological replicates. *p < 0.05; **p < 0.01 (two-way ANOVA). The immunoblot data shown in E-F were representative repeats from two independent biological experiments. KO#1 and KO#2 are two individual clones generated for *GSDMB* knockout after CRISPR/Cas-9 editing.

Additionally, stable knockout (KO) of *GSDMB* by CRISPR-Cas9 editing dampened induction of *ISGs* as well as *IFNs* in BEAS-2B cells transfected with poly (dA:dT) (Figure 2A–D). Furthermore, the phosphorylated TBK1/IRF3 and phosphorylation-dependent dimerization of IRF3 were also subdued in *GSDMB* KO cells (Figure 2E,F), indicating that GSDMB is indeed required by the double stranded DNA such as poly (dA:dT)-induced activation of cGAS-STING pathway, as well as subsequent induction of *IFNs* and *ISGs*.

### 3.4. GSDMB Facilitates mtDNA-induced IFN Response and ISGs Activation

Cell free mtDNA, one major source of double stranded DNA that cells may expose to, has been detected in plasma samples or bronchoalveolar lavage fluid (BALF) from patients with asthma [45,67,68]. We, therefore, extracted and quantified the cell free mtDNA in asthmatic plasma samples (*n* = 357) from the Costa Rico asthma cohort (GACRS) [69], a relatively mild asthmatic cohort (Table S4).

**Figure 3.**
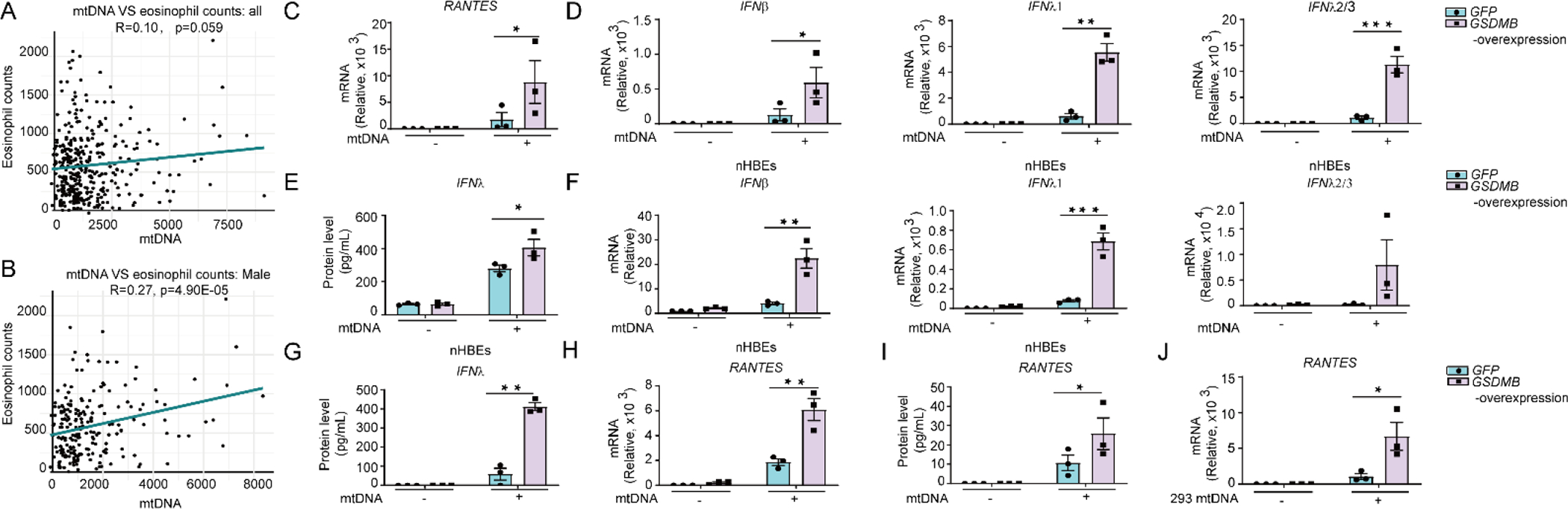
GSDMB promotes mtDNA-induced *ISG* expression. (**A**) Correlation analysis between mtDNA copy number in plasma samples and eosinophil counts in plasma samples from asthmatic individuals from Costa Rico asthma cohort (GACRS). (**B**) Male subjects were included for the correlation analysis in A. (**C–E**) The mRNA levels of *RANTES* (**C**), *IFNβ*, *IFNλ1* and *IFNλ2/3* (**D**) as well as the protein level of IFNλ (**E**) were measured in BEAS-2B cells with overexpression of *GSDMB* and transfection of mitochondrial DNA (mtDNA) extracted from BEAS-2B cells for 12 h. (**F–I**) The mRNA (**F**) or protein levels of IFNs (**G**), as well as the mRNA (**H**) or protein levels (**I**) of RANTES were assessed in nHBEs with stable overexpression of *GSDMB* and transfected with mtDNA extracted from BEAS-2B cells for 12 h. (**J**) Expression of *RANTES* was measured in BEAS-2B cells with overexpression of *GSDMB* and transfection of HEK 293 cells-derived mtDNA for 12 h. Means ± SEM shown in C-J were from three independent biological replicates. *p < 0.05; **p < 0.01 (two-way ANOVA).

We then compared the correlation of mtDNA levels with multiple clinical characteristics in asthmatics. With limited sample size, we found that mtDNA copy number significantly correlates with eosinophil counts (Figure 3A), mainly in the male, but not female asthmatic samples (Figures 3B and S4A).

**Figure 4.**
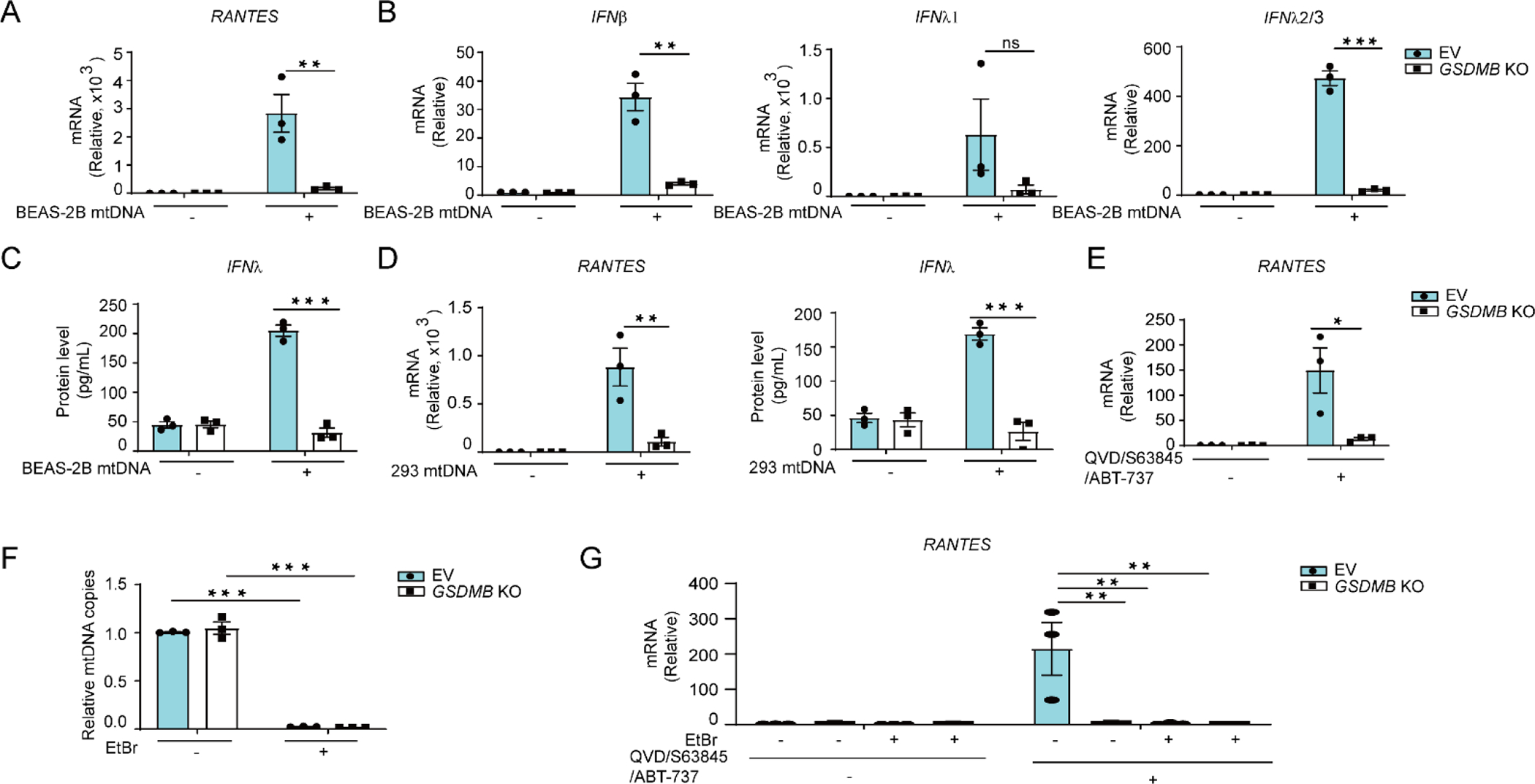
*GSDMB* deficiency impairs mtDNA-induced *ISGs* expression. (**A–C**) BEAS-2B cells with or without knockout (KO) of GSDMB were transfected with mtDNA extracted from BEAS-2B for 12 h, and the mRNA levels of *RANTES* (**A**), *IFNβ*, *IFNλ1* and *IFNλ2/3* (**B**) or IFNλ protein levels (**C**) were then determined. (**D**) The mRNA levels of *RANTES* or protein levels of *IFNλ* were measured in empty vector (EV)-transfected or *GSDMB* KO BEAS-2B cells which were transfected with 293 mtDNA for 12 h. (**E**) Expression of *RANTES* was measured in EV or *GSDMB* KO BEAS-2B cells treated with ABT-737 (20 μM), S63845 (20 μM) and QVD-OPh (20 μM) for 3 h. (**F**) The mtDNA amount in total DNA extracts measured by qPCR in EV or *GSDMB* KO BEAS-2B cells, treated with or without ethidium bromide (EtBr) for 7 days. (**G**) The expression of *RANTES* in EV or *GSDMB* KO BEAS-2B cells depleted of mtDNA followed by treatment with ABT-737 (20 μM), S63845 (20 μM) and QVD-OPh (20 μM) for 3 h. Means ± SEM shown are from three independent biological replicates. *, *p* < 0.05; **, *p* < 0.01 (two-way ANOVA).

We therefore set out to determine whether GSDMB regulates mtDNA-induced IFN response and *ISGs* expression [44]. Firstly, we extracted mtDNA from the bronchial epithelial BEAS-2B cell line and transfected them into *GSDMB*-overexpressing cells. Similar as poly (dA:dT), transfected BEAS-2B mtDNA induced robust *ISGs* and *IFNs* production, which was enhanced by overexpression of *GSDMB* in both BEAS-2B cells (Figures 3C–E and S4B) and nHBEs cells (Figure 3F,I). Furthermore, mtDNA extracted from HEK293 cells also activated significant induction of *ISGs* and *IFNs*, especially in *GSDMB*-overexpressing cells (Figures 3J and S4C). Conversely, knockout of *GSDMB* alleviated mtDNA-induced IFN production and *ISGs* expression in BEAS-2B cells (Figures 4A–D and S5A–C).

In addition to exogenously transfected mtDNA, we also attempted to induce release of cellular mtDNA by various stimulation. We first employed IL-1β, a pleiotropic inflammatory cytokine that was reported to promote mtDNA release in cancer cell line and immune cells [70]. However, no remarkable mtDNA release was detected in BEAS-2B cells treated with IL-1β (Figure S6). As an alternative approach, we treated cells with QVD-OPh, S63845 and ABT-737 by combined inhibition on pan-caspase, myeloid leukemia 1 (MCL-1) and B-cell lymphoma xL (BCL-xL)/BCL-2/BCL-w respectively, as previously reported for inducing mitochondrial apoptosis and subsequent mtDNA release in cancer cells and primary mouse embryonic fibroblasts (MEFs) [50–52,71,72]. Indeed, we observe significant mtDNA release in bronchial epithelial BEAS-2B cells in response to the combined treatment of QVD-OPh, S63845, and ABT-737 (Figure S6). The mtDNA release results in *RANTES* induction in a cGAS-STING pathway-dependent manner (Figure S7). Furthermore, deficiency of *GSDMB* significantly impaired induction of *RANTES* by released endogenous mtDNA (Figure 4E) in KO cells despite comparable amount of released mtDNA in both WT and KO (Figure 4F). Most importantly, depletion of mtDNA by prolonged treatment with ethidium bromide (EtBr) results in significant reduction of *RANTES* induction in both WT and *GSDMB* KO cells with combined treatment of QVD-OPh/S63845/ABT-737 in (Figure 4F,G), indicating that induction of *RANTES* by QVD-OPh/S63845/ABT-737 depends on mtDNA release. Taken together, these findings suggest that GSDMB promotes mtDNA-induced IFNs production and *ISGs* expression.

### 3.5. GSDMB Promotes dsDNA-induced ISGs Expression and IFN Production via the cGAS-STING Pathway

**Figure 5.**
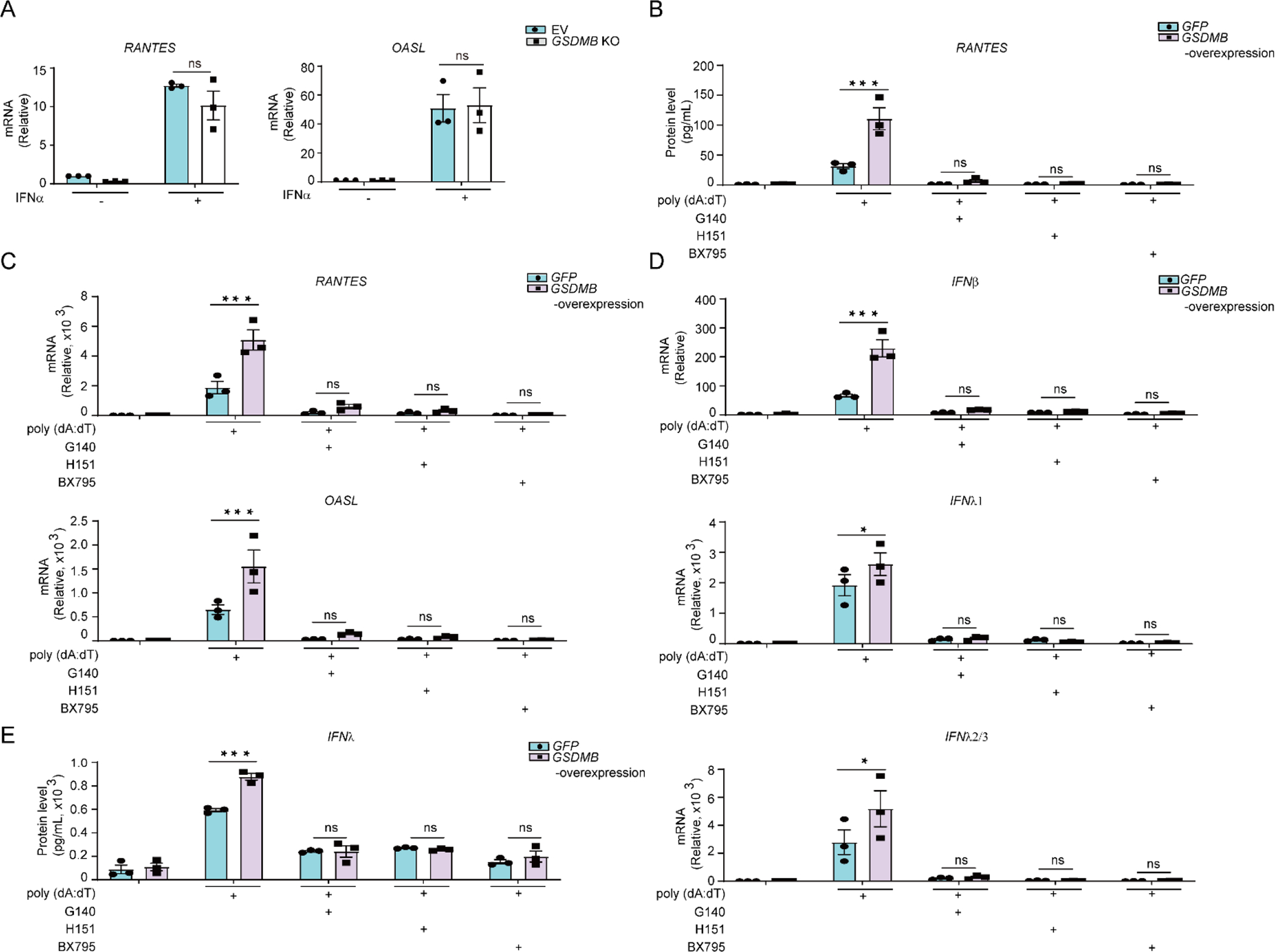
GSDMB promotes dsDNA-induced *ISGs* expression and IFN production through the cGAS-STING pathway. (**A**) Expression of *RANTES* and *OASL* in empty vector (EV)-transfected or *GSDMB* KO BEAS-2B cells treated with IFNα (500 U/mL) for 24 h. (**B**) Secreted RANTEs protein was measured in the supernatant from GFP- or *GSDMB*-overexpressing BEAS-2B cells pretreated with various inhibitors for the cGAS/STING pathway: G140 (40 μM), H151 (20 μM) or BX795 (10 μM) for 1 h followed by transfection with poly (dA:dT) for 6 h. (**C**) Expression of *RANTES* and *OASL* measured in cells as described in B. (**D**) Expression of *IFNβ*, *IFNλ1*, *IFNλ2/3* measured in cells as described in **B**. (**E**) Protein levels of IFNλ were measured in supernatant from cells described in **B.** Data are presented as means ± SEM from three independent biological replicates. *p < 0.05; **p < 0.01 (two-way ANOVA).

To gain insight into the mechanisms of how GSDMB regulates dsDNA-induced *ISGs* expression, we treated cells with IFNα, which leads to comparable induction of *ISGs* in *GSDMB* KO cells and control WT cells, indicating that GSDMB likely functions upstream instead of downstream of IFNs (Figure 5A). TBK1 is a central hub for inducing IFN production. BX795, a synthetic inhibitor of TBK1 [73], effectively blocked *IFN* production and *ISGs* expression in both control and *GSDMB*-overexpressing cells in response to transfection of poly (dA:dT) (Figure 5B–E). Similarly, G140 and H151, two potent and selective inhibitors of cyclic GMP-AMP synthase (cGAS) and STING, respectively [74,75], significantly mitigated GSDMB-enhanced *ISGs* expression and IFN response upon poly (dA:dT) stimulation (Figure 5B–E). These data indicate that GSDMB functions via the cGAS-STING-TBK1 pathway in promoting *ISGs* expression and *IFN* production induced by dsDNA.

### 3.6. GSDMB Interacts with STING and Accelerates STING’s Translocation from ER to Golgi

**Figure 6.**
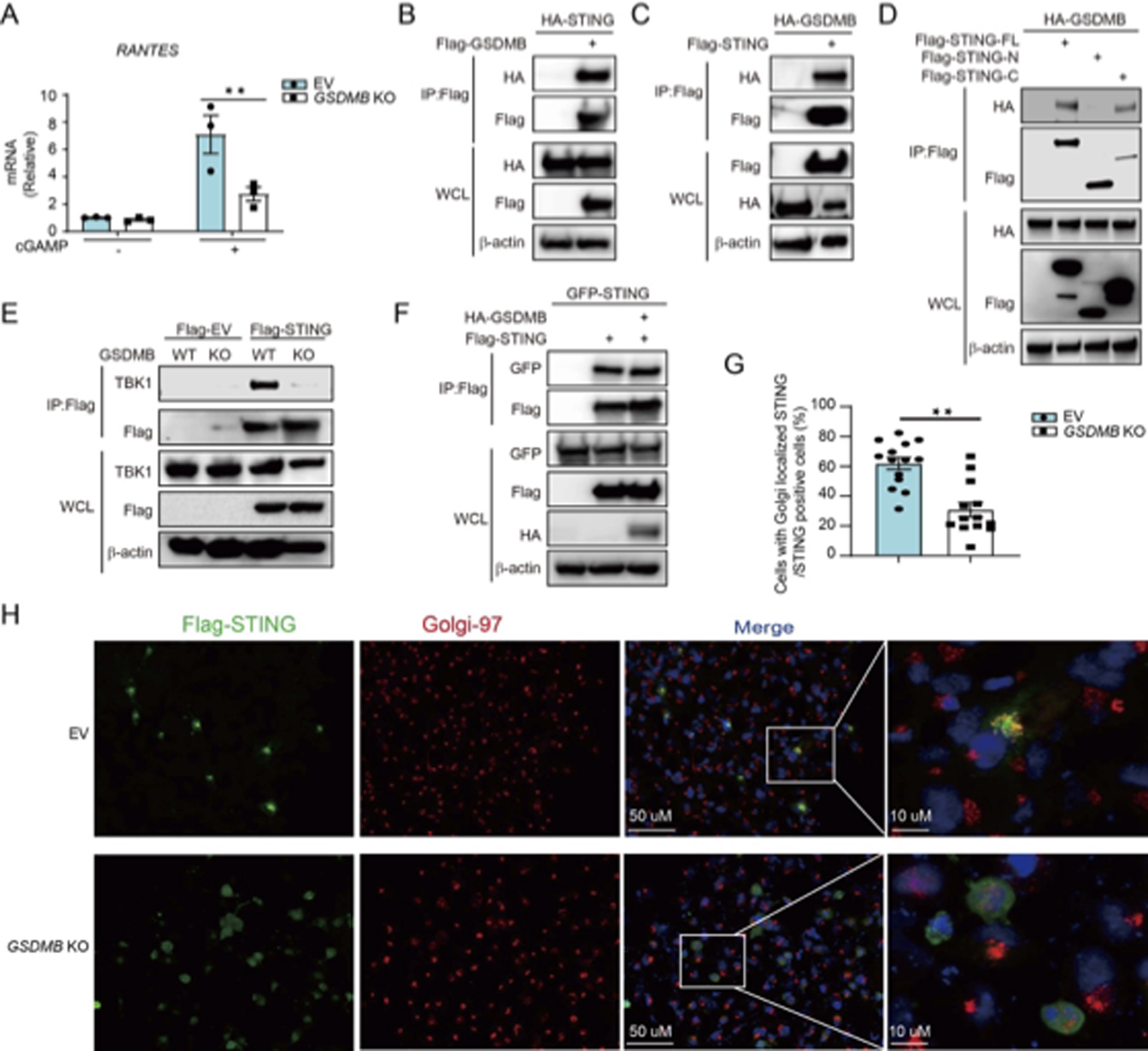
GSDMB interacts with STING and promotes its translocation into Golgi. (**A**) Expression of RANTES in empty vector (EV)-transfected or *GSDMB* KO BEAS-2B treated with cGAMP. (**B–D**) Immunoprecipitation assay (IP) using anti-FLAG beads and immunoblotting with HA antibody in HEK293 cells transfected with FLAG-GSDMB and HA-STING (**B**) or FLAG-STING and HA-GSDMB (**C**) or FLAG-STING at various lengths (i.e., deletion mutants of STING) and HA-GSDMB (**D**), respectively. (**E**) IP assay using anti-FLAG beads in EV or *GSDMB* KO BEAS-2B cells transfected with Flag-STING and the proteins were further analyzed by immunoblotting with anti-TBK1 antibody. (**F**) Immunoprecipitation assay (IP) using anti-FLAG beads followed by immunoblotting using anti-GFP antibody in HEK293 cells co-transfected with Flag-STING, HA-GSDMB and GFP-STING to evaluate the dimerization of STING. (**G–H**) Quantification (**G**) on immunofluorescence staining (**H**) of Flag-tagged STING (green) and Golgi tracker Golgi-97 (Cell Signaling Technology) (red) in EV or *GSDMB* KO BEAS-2B cells transfected with Flag-STING for 48 h followed by transfection with poly (dA:dT) for 6 h. (**G**). The immunoblotting data shown are representative of two independent biological experiments. Each dot in G represents one independent view from total more than 12 independent views from four repeats in BEAS-2B cells.

Having positioned GSDMB in the cGAS-STING pathway, we next sought to explore the target of GSDMB in this pathway. Firstly, treatment of the WT and *GSDMB* KO BEAS-2B cells with cGAMP, the second messenger synthesized by cGAS and recognized by STING [18,76] failed to restore the induction of *RANTES* in *GSDMB* KO cells, indicating that GSDMB potentially works downstream of cGAS (Figure 6A). Secondly, co-immunoprecipitation and immunoblot analyses showed that GSDMB interacts with STING, possibly through the C-terminal domain of STING (Figure 6B–D). Importantly, *GSDMB* KO cells demonstrated reduced interaction between STING and TBK1, suggesting that GSDMB likely facilitates the interaction of STING with TBK1 without influencing the dimerization of STING (Figure 6E,F). Additionally, as the interaction of STING and TBK1 occurs in Golgi, we next examined the location of STING under *GSDMB* deficiency [77–80]. Indeed, *GSDMB* KO led to reduced localization of STING into the Golgi (Figure 6G,H).

Overall, GSDMB promotes mtDNA- and poly (dA:dT)-induced activation of cGAS-STING pathway through interacting and promoting the translocation of STING to Golgi and subsequent association with TBK1, activating IFN production and induction of *ISGs*.

## 4. Discussion

In our study, we found that GSDMB, the asthma risk gene located in the 17q21, promotes the activation of mtDNA-induced cGAS-STING pathway via facilitating the translocation of STING into Golgi and subsequent interaction with TBK1. Consistent with this, overexpression of GSDMB led to increased, while deficiency of *GSDMB* led to impaired *ISGs* expression in normal human bronchial epithelial cells or bronchial epithelial cell lines induced by either transfected mtDNA or endogenously released mtDNA. In addition, chemical inhibition of cGAS by G140, STING by H151 treatment, or TBK1 by BX795 effectively attenuated induction of IFNs and *ISGs*, suggesting that targeting these signaling molecules may provide a novel therapeutic strategy for airway inflammation in asthmatics, especially for patients who carry the risk alleles at 17q21.

Mitochondria, an important organelle, have been linked to the development of asthma with increased levels of ATP content, mitochondrial respiration, mitochondrial mass, reactive oxygen species (ROS), and mitochondrial arginine metabolism detected in asthmatic patients [81–84]. Additionally, mtDNA has attracted increasing attention in asthma research recently [85]. Alternaria extract and house dust mite extract robustly induced mtDNA release from airway epithelial cells, amplifying type 2 immune response in airways in both cellular and mouse models [46,86]. Perfluoroalkyl substances (PFAS), a commonly used chemical compound in manufacturing processes, promote mtDNA release and enhance subsequent inflammation through AIM2-mediated inflammasome activation, ultimately resulting in aggravated inflammation in mouse models. [43]. These findings support an important role of mtDNA in asthma development.

However, roles of mtDNA in human asthmatic samples have been controversial. We found a positive correlation between mtDNA and eosinophil counts in asthmatic plasma samples, consistent with previously reported higher mtDNA levels in asthma samples/models [43,45,67,68,86]. However, asthmatic patients with high fractional exhaled nitric oxide (FeNO) were reported having lower mtDNA levels in bronchoalveolar lavage fluid [45]. Such discrepancy likely results from various endotypes of asthma included in each study given that distinct inflammatory pathways related to eosinophil counts and FeNO are weakly correlated and have different outcomes in large clinical trials in asthma [87,88]. In addition, disease severity, genotypes and genders of asthmatic subjects also need to be considered for data interpretation in each study, given that we observed stronger correlation between mtDNA and eosinophil counts only in the male group. Thus, further investigation on mtDNA levels in a larger cohort with well-characterized clinical endo-types of asthma is necessary to determine roles of mtDNA in asthma, especially the mechanism underlying gender difference.

GSDMB regulates inflammasome-induced pyroptosis, similar to its paralogs such as gasdermins A and D [11,60]. The inflammasome, a multi-protein complex responsible for inflammation and cell death, plays a significant role in the onset of various respiratory diseases, including chronic rhinosinusitis (CRS) [64], chronic obstructive pulmonary disease (COPD) [89] and asthma [43]. While the function of GSDMB in anti-tumor immunity through pyroptosis is well characterized [11,60], GSDMB was also reported to have pyroptosis-independent function such as its regulation of PDGF-A-mediated FAK phosphorylation [14], MAVS-TBK1 signaling [90] and 5-lipoxygenase (5-LO)-mediated TGF-β1 expression [13]. Here, we reported another pyroptosis-independent function of GSDMB in human airway epithelial cells by positively regulating the cGAS-STING pathway, leading to a downstream *ISGs* induction as well as inflammation. Therefore, such pyroptosis-independent function is exclusive to GSDMB, in contrast to GSDMD.

### Limitations of the study

Admittedly, there are several limitations in this study. First, while we observed significant correlation between mtDNA and eosinophil counts only in male asthmatic subjects, which is consistent with the higher prevalence and more severity of asthma in boys compared to girls. However, the underlying mechanism for gender-specific effects requires further studies. Secondly, the in vivo significance of regulation of GSDMB on mtDNA-induced activation of the cGAS-STING pathway needs additional research in the future. Lastly, future investigation is warranted to determine how GSDMB promotes the translocation of STING from ER to Golgi.

## 5. Conclusions

In summary, we discovered a novel role for GSDMB, a genetic risk factor for asthma associated with 17q21 locus in regulating mtDNA-induced innate immune response in respiratory epithelial cells. Specifically, we found that GSDMB promotes the induction of interferons and subsequent activation downstream *ISGs* via the cGAS-STING pathway in response to mtDNA. Thus, these findings suggest that GSDMB may have the therapeutic potential for treating asthma especially in genetically susceptible subjects.

## Supplementary Material

Supplementary Information
