# Supplementary material for: The Asthma Risk Gene, GSDMB, Promotes Mitochondrial DNA-induced ISGs Expression
Source: J Respir Biol Transl Med. Author manuscript; Available in PMC 2024 May 10. (PMC11086750; doi:10.35534/jrbtm.2024.10005)
Supplement: Supplementary Information [file NIHMS1982596-supplement-Supplementary_Information.pdf]

Supplemental Material

The Asthma Risk Gene, GSDMB, Promotes Mitochondrial DNA-induced *ISGs* Expression

Tao Liu, Julian Hecker, Siqi Liu, Xianliang Rui, Nathan Boyer, Jennifer Wang, Yuzhen Yu, Yihan Zhang, Hongmei Mou, Luis Guillermo Gomez-Escobar, Augustine M.K. Choi, Benjamin A. Raby, Scott T. Weiss, Xiaobo Zhou *

Methods

Transfection of poly (dA:dT) or mtDNA in Cells

Transfection mixture of 2 µg/mL of ploy (dA:dT) or mtDNA with Lipofectamine™ 3000 was prepared in Opti-MEM® Reduced Serum Medium (Life Technologies) Transfection Reagent (Life Technologies). Then the poly (dA:dT) or mtDNA mixture was added into the BEAS-2B cells in a 6-well plate at 90% confluency. Six hours post-transfection, cells were collected for qPCR or Western Blotting analysis. Additionally, the supernatant was collected for ELISA measurements using the Human RANTES ELISA Kits (Fisher Scientific), IFN beta Human ELISA Kit (Fisher Scientific), or Human IL-29/IL-28B (IFN-lambda 1/3) DuoSet ELISA kit (R&D Systems) following the manufacturer's protocol.

mtDNA Extraction and Quantification from Plasma

Plasma samples (n = 375) from asthmatic subjects were thawed on ice and mixed briefly using a vortex mixer. Next, 50 µL of plasma was combined with 170 µL PBS and mixed thoroughly. The diluted sample was then centrifuged for 5 min at 4 degrees at 700 g. After centrifugation, 200 µL of supernatant was collected and further centrifuged for 15 min at 4 degrees at 18,000 g. The collected supernatants were used for DNA extraction and quantification as previously described [1], using the QIAamp DSP DNA Blood Mini Kit (Qiagen, Cat. 61104). The quantity of mtDNA was then determined by qPCR, using Power SYBR Green PCR Master Mix (Thermo Fisher Scientific). The primer sequences were provided in Table S2. The linear dsDNA standard was synthesized from IDT to measure copy number of human mitochondrially encoded NADH dehydrogenase 1 (MTND1). The sequence is: 5’-AACAACATACCCATGGCCAACCTCCTACTCCTCATTGTACCCATTCTAATCGCAATGGCATTCCTAATGC TTACCGAACGAAAAATTCTAGGCTATATACAACTACGCAAAGGCCCCAACGTTGT -3’.

Quantitative RT-PCR

Total RNA was extracted using the Direct-zol™ RNA MiniPrep with TRI-Reagent® (Zymo Research) following the manufacturer's instructions. To perform the RT-PCR analysis, cDNA was synthesized using the High-Capacity cDNA Reverse Transcription Kit with RNase and then gene expression was measured by either Applied Biosystems Power SYBR Green PCR Master Mix (Thermo Fisher Scientific) or TaqMan™ Fast Advanced Master Mix (LIFE TECHNOLOGIES CORPORATION). The expression levels of all genes were normalized to GAPDH. human *GAPDH* (Hs.PT.39a.22214836) and human *XBP1* (Hs.PT.58.1903847) probes are purchased from Integrated DNA Technologies IDT. Primer sequences were provided in Table S2.

Immunofluorescence Staining

Cells grown on CultureSlides (Corning) were fixed with 4% paraformaldehyde solution in PBS (ChemCruz) then permeabilized with ice-cold 100% methanol, blocked with blocking buffer (1X PBS/5% Goat serum/0.3% Triton X-100) and incubated with various primary antibodies at 4 °C overnight including Anti-Flag antibody (Sigma-Aldrich), Golgin-97 Antibody (Cell Signaling Technology). Following incubation with Goat anti-Mouse IgG/IgM (H+L) Secondary Antibody, Alexa Fluor 488 (Thermo Fisher) and Donkey anti-Rabbit IgG (H+L) Secondary Antibody, Alexa Fluor® 647 conjugate (Invitrogen) for 1 h at room temperature, they were counter-stained with DAPI in Prolong™ Gold antifade reagent (Invitrogen). The images were captured and examined using ZEISS Axio Imager 2.

Immunoprecipitation and Immunoblot Analysis

https://doi.org/10.35534/jrbtm.2024.10005

For immunoprecipitation, total cellular protein was extracted from cells transfected with plasmids or poly (dA:dT). The extracts were then lysed in low-salt lysis buffer (50 mM HEPES, 150 mM NaCl, 1 mM EDTA, 1.5 mM $MgCl_2$, 10% glycerol, 1% Triton X-100), and incubated overnight with anti-FLAG beads (Sigma-Aldrich). The beads were washed three to five times with low-salt lysis buffer, and the immunoprecipitates were washed then loaded with 4x Laemmli Sample Buffer (BioRad) and separated by pre-cast 5-12% SDS-PAGE gel (Bio-Rad, #4561093 and #4561096). The proteins were then transferred to polyvinylidene fluoride (PVDF) membranes (Bio-Rad) and further probed with the relevant antibodies listed in Table S1. Proteins was detected by Thermo Scientific SuperSignal West Femto Chemiluminescent Substrate (Thermo Fisher Scientific) or Thermo Scientific Pierce ECL Western Blotting Substrate (Thermo Fisher Scientific) and images were captured using the G:Box system (Syngene).

Table S1. Antibodies used in Western Blotting.

REAGENT or RESOURCE	SOURCE
Rabbit monoclonal anti-TBK1 antibody	Abcam (ab40676)
Rabbit monoclonal anti-Phospho-TBK1 antibody	Cell Signaling Technology (5483S)
Rabbit monoclonal Anti-IRF3 antibody	Abcam (ab68481)
Rabbit monoclonal Anti-Phospho-IRF3 antibody	Cell Signaling Technology (4947S)
Chicken polyclonal Anti-GFP antibody	Abcam (ab13970)
Mouse monoclonal HRP Anti-beta Actin antibody	Abcam (ab49900)
Rabbit monoclonal antibody Anti- DYKDDDDK Tag	Cell Signaling Technology (14793S)
Anti-FLAG® Affinity Isolated Mouse Monoclonal Antibody [M2]	Sigma-Aldrich (F1804-200UG)
Anti-HA antibody produced in rabbit	Sigma-Aldrich (H6908-100UL)
Goat polyclonal anti-Mouse IgG(H+L) Secondary Antibody	Novus Biologicals (NBP1-75957)
Donkey polyclonal anti-Rabbit IgG (H+L) Highly	Thermo Fisher Scientific (31458)
Agarose Gel Anti-FLAG® Mouse Monoclonal Antibody [M2]	Sigma-Aldrich (A2220-5ML)
Anti-ds DNA antibody	Abcam (ab27156)
Golgin-97 (D8P2K) Rabbit mAb	Cell Signaling Technology (13192S)

Table S2. Primers used in RT-PCR assays.

Gene Name	Primers
human IFNβ	sense: 5’-ATGACCAACAAGTGTCTCCTCC-3’
	anti-sense:5’-GGAATCCAAGCAAGTTGTAGCTC-3’
human IFNλ1	sense: 5’-CACATTGGCAGGTTCAAATCTCT-3’
	anti-sense:5’-CCAGCGGACTCCTTTTTGG-3’
human IFNλ2/3	sense: 5’-CTGCCACATAGCCCAGTTCA-3’
	anti-sense:5’-AGAAGCGACTCTTCTAAGGCATCTT-3’
human RANTES	Forward 5’-CCAGCAGTCGTCTTTGTCAC-3’
	Reverse 5’-CTCTGGGTTGGCACACACTT-3’
human OASL	sense: 5’-CTGATGCAGGAACTGTATAGCAC-3’
	anti-sense:5’-CACAGCGTCTAGCACCTCTT-3’
Human GAPDH	sense: 5’-GTCGCTGTTGAAGTCAGAGG-3’
	anti-sense:5’-GAAACTGTGGCGTGATGG-3’
Human D-LOOP	sense: 5’-GTTTATGTAGCTTACCTCCTC-3’
	anti-sense:5’-TTGTTTATGGGGTGATGTGAG-3’
human CHOP	sense: 5’-GCACCTCCCAGAGCCCTCACTCTCC-3’
	anti-sense:5’-GTCTACTCCAAGCCTTCCCCCTGCG-3’
human BIP	sense: 5’-CGAGGAGGAGGACAAGAAGG-3’
	anti-sense:5’-CACCTTGAACGGCAAGAACT-3’
human XBP1s	sense: 5’-TGCTGAGTCCGCAGCAGGTG-3’
	anti-sense:5’-GCTGGCAGGCTCTGGGGAAG-3’

Native Polyacrylamide Gel Electrophoresis (PAGE) Analysis of Oligomers

IRF3 oligomers and dimers were detected using native PAGE in BEASE-2B cells with stable overexpression or knockout of GSDMB. Briefly, cellular protein extracts were obtained from BEAS-2B cells after transfection with poly

(dA:dT) at designated time points using a low-salt lysis buffer (50 mM HEPES, 150 mM NaCl, 1 mM EDTA, 1.5 mM $MgCl_2$, 10% glycerol, 1% Triton X-100). The samples were then mixed with Native Sample Buffer (Bio-Rad) and separated on a pre-cast 4–20% Mini-PROTEAN TGX Gel (Bio-Rad), using TGS buffer (Thermo Fisher Scientific) for 30 min before the proteins were transferred to PVDF membranes (Bio-Rad) and detected with the corresponding antibodies including IRF3, STING, GFP and TBK1 (Table S1) by Western Blotting as described above.

Table S3. Differential correlation analysis of *GSDMB* with various interferon stimulated genes (ISGs) in asthmatic and control samples from Genes-environments and Admixture in Latino Americans (GALA) II study.

	**Gene1**	**Gene2**	**Cor_cases**	**Cor_controls**	**Diff_Cor_*p*-value**
01	***GSDMB***	***RANTES***	**0.5063546**	**0.2816500**	**0.0003**
02	*GSDMB*	*CCL8*	0.4097280	0.4657069	0.5276
03	*GSDMB*	*CXCL9*	0.5941375	0.4987806	0.1505
04	*GSDMB*	*CXCL10*	0.5975816	0.5145237	0.2146
05	*GSDMB*	*ISG15*	0.4938389	0.5096487	0.8395
06	*GSDMB*	*ISG20*	0.3643795	0.4412306	0.4335
07	*GSDMB*	*GBP1*	0.6006391	0.5566749	0.4935
08	*GSDMB*	*GBP2*	0.5733306	0.5521058	0.7503
09	*GSDMB*	*IRF1*	0.6297007	0.5733622	0.3546
10	*GSDMB*	*IRF7*	0.4019199	0.4365557	0.7119
11	*GSDMB*	*IFNγ*	0.5582008	0.4986320	0.3770
12	*GSDMB*	*IFNλ2*	0.3385870	0.3564360	0.8400

Significant differential correlation was indicated by bold for RANTES; *Cor: Correlation.

Table S4. Demographics (mean±SDs) of subjects in Costa Rica asthma cohort.

	**Total**	**Male**	**Female**
No. subject	375	229	146
Age (year)	8.7 ± 1.4	8.7 ± 1.5	8.7 ± 1.3
Fev1_fvc_bd0	82.0 ± 7.0	82.1 ± 7.0	81.9 ± 6.9
pctpred_fev1_pre_BD	97.7 ± 17.7	99.5 ± 16.5	95.0 ± 19.3
Total serum IgE (IU/mL)	696.7 ± 858.4	745.7 ± 933.1	620.0 ± 722.5
Blood eosinophils (cells/mL)	584.4 ± 428.1	562.6 ± 393.4	618.7 ± 477.0

Fev1_fvc_bd0: FEV1/FVC Pre-broncodilator; pctpred_fev1_pre_BD: % predicted Pre-BD FEV1 [(PreFEV/FEVpred) *100].

Cytotoxicity Assay

After cells were transfected with poly (dA:dT) for 6 h, supernatants were collected and the level of cell death was then determined by measurements of the lactate dehydrogenase (LDH) release using the CytoTox 96 Non-Radioactive Cytotoxicity Assay kit (Promega) based on the manufacture's protocol.

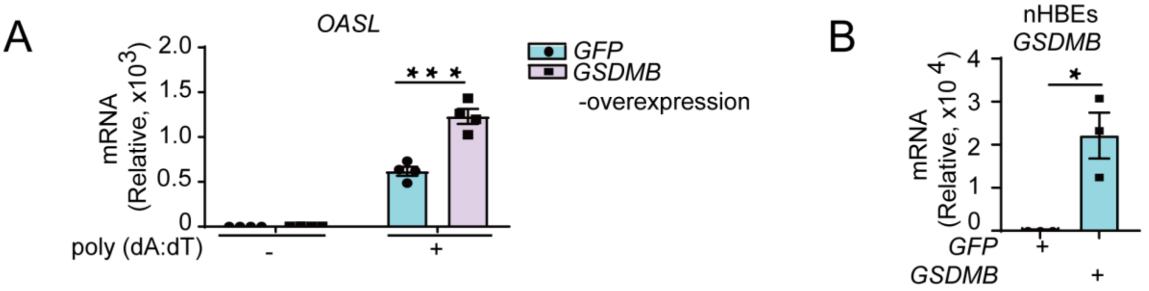

Figure S1. GSDMB promotes dsDNA-induced *ISG* expression. (**A**) The mRNA level of *OASL* was measured in BEAS-2B cells transfected with poly (dA:dT) for 6 h. Both control cells stably transfected with GFP and GSDMB-overexpressing cells were used. (**B**) Expression of *GSDMB* in nHBEs with stable overexpression of GSDMB as assessed by qPCR. Mean ± SEMs shown from at least three independent biological replicates. *$p < 0.05$; **$p < 0.01$ (two-way ANOVA).

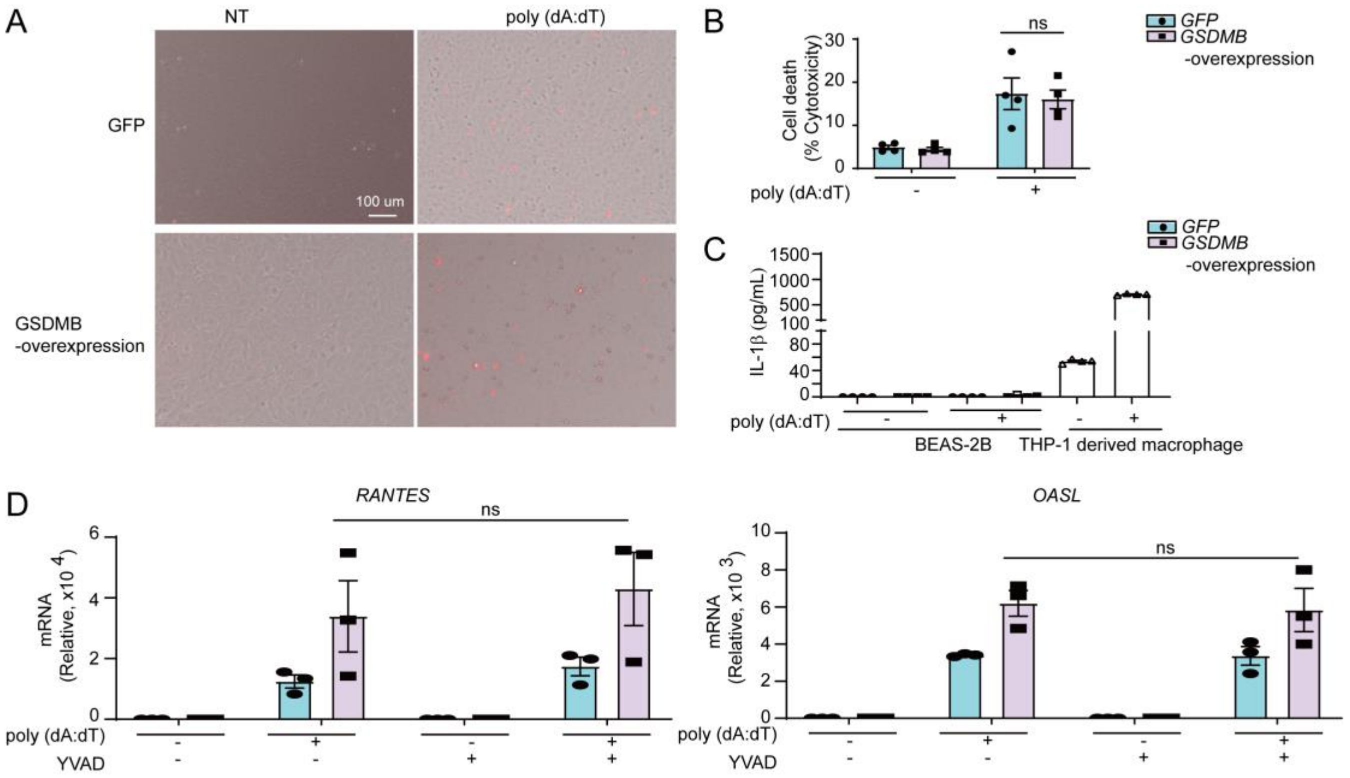

Figure S2. Overexpression of GSDMB showed no impacts on dsDNA-induced cell death. (**A**) Phase contrast images of BEAS-2B cells with or without overexpression of GSDMB after transfection with poly (dA:dT) (2 µg/mL) for 6 h. Cells in red color have positive staining with propidium iodide (PI) indicative of dead cells. (**B**) Cell death measurements by lactate dehydrogenase (LDH) release culture supernatant in BEAS-2B cells transfected with poly (dA:dT) for 6 h. (**C**) Measurements of IL-1β in supernatant from BEAS-2B cells with or without GSDMB overexpression transfected with poly (dA:dT) (2 µg/mL) for 6 h. THP-1 cells stimulated with phorbol 12-myristate 13-acetate (PMA) (100 ng/mL) overnight followed by subsequent transfection of poly (dA:dT) (2 µg/mL) for 6 h were used as a positive control for IL-1β secretion. (**D**) Expression of RANTES (Left) and OASL (Right) in BEAS-2B cells, with or without overexpression of GSDMB and transfected with poly (dA:dT) (2 µg/mL) for 6 h with or without pretreatment of YVAD (50 µM) for 1 h. Error bars represent standard errors of mean(SEM) from four independent biological replicates. *$p < 0.05$; **$p < 0.01$ (two-way ANOVA).

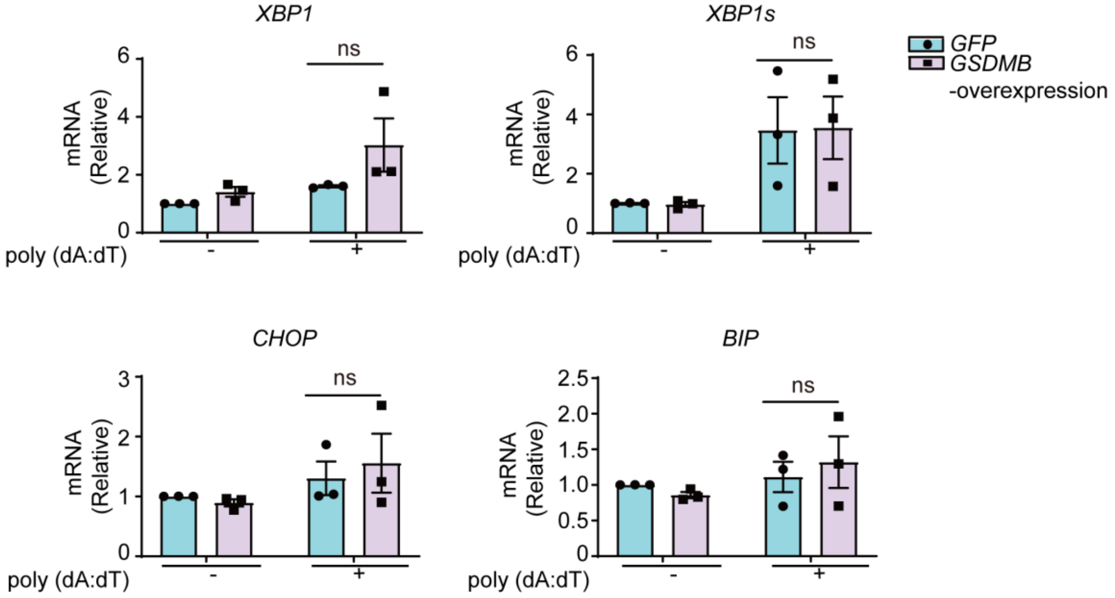

Figure S3. GSDMB promotes cGAS-STING signaling independent of ER stress. Expression of BIP, CHOP, XBP1, and splicing XBP1 (XBP1s) measured in BEAS-2B cells overexpressing GFP or GSDMB transfected with or without poly (dA:dT) for 6 h. Mean ± SEMs shown from at least three independent biological replicates. *$p < 0.05$ (two-way ANOVA).

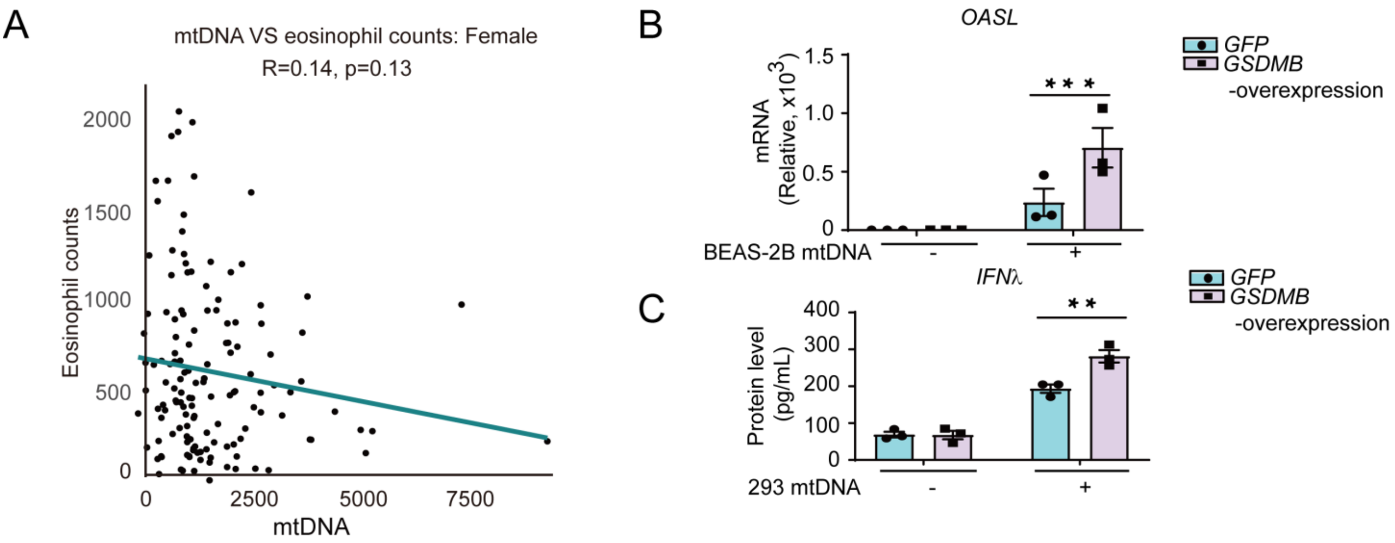

Figure S4. GSDMB elicits mtDNA-induced interferon response. (**A**) Female subjects were included for the correlation analysis between mtDNA copy number in plasma samples and eosinophil counts in plasma samples from asthmatic individuals from Costa Rico asthma cohort (GACRS). (**B**) Expression of *OASL* was measured in BEAS-2B cells with overexpression of GSDMB after transfection of mtDNA derived from BEAS-2B cells for 12 h. (**C**) Expression of *IFNλ* was measured in BEAS-2B cells with overexpression of GSDMB after transfection of mtDNA extracted from HEK 293 cells for 12 h.

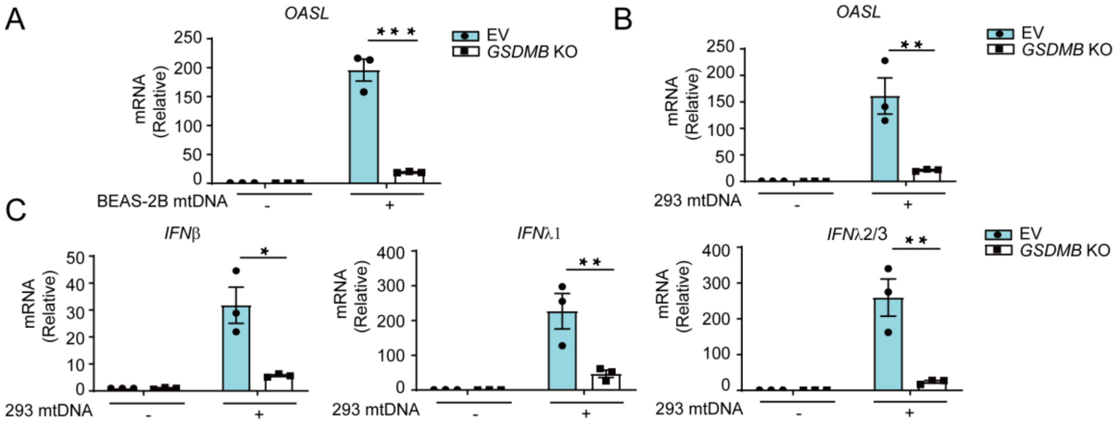

Figure S5. Deficient GSDMB attenuates mtDNA-induced IFN response. (**A**) The mRNA level of *OASL* was determined in BEAS-2B cells with GSDMB overexpression and transfection of BEAS-2B mtDNA for 12 hr. (**B-C**) The mRNA expression levels of *OASL* (B), *IFNβ*, *IFNλ1* and *IFNλ2/3* (C) were assessed in BEAS-2B cells with overexpression of GSDMB after transfection of mtDNA derived from 293 cells for 12 h. Mean ± SEMs from three independent biological replicates. *$p < 0.05$; **$p < 0.01$ (two-way ANOVA).

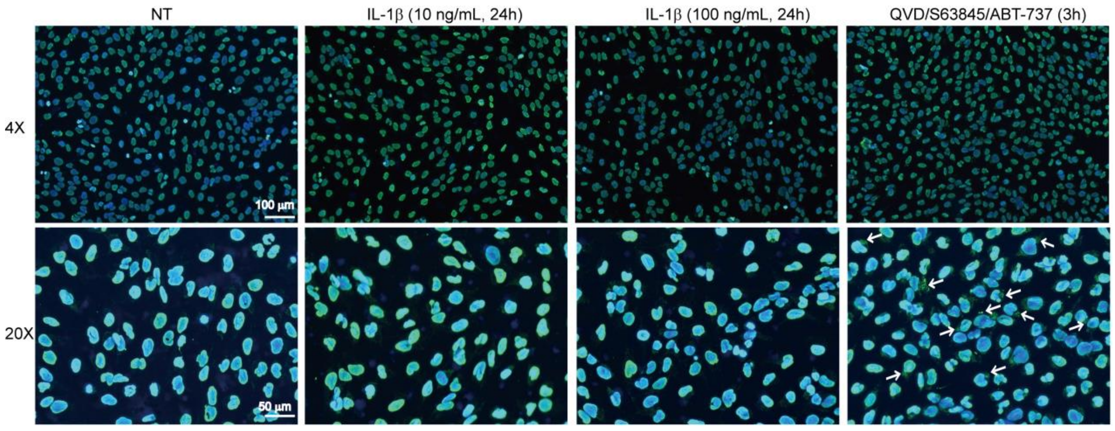

Figure S6. Mitochondrial apoptosis induces mtDNA release in epithelial cells. Immunostaining with antibodies for dsDNA (green) and nuclei counterstained with DAPI (blue) in BEAS-2B treated with IL-1β (10 ng/mL or 100 ng/mL) for 24 h or treated with ABT-737 (20 µM), S63845 (20 µM) and qVD-OPh (20 µM) for three h. Scale bar = 50 µm or 100 µm.

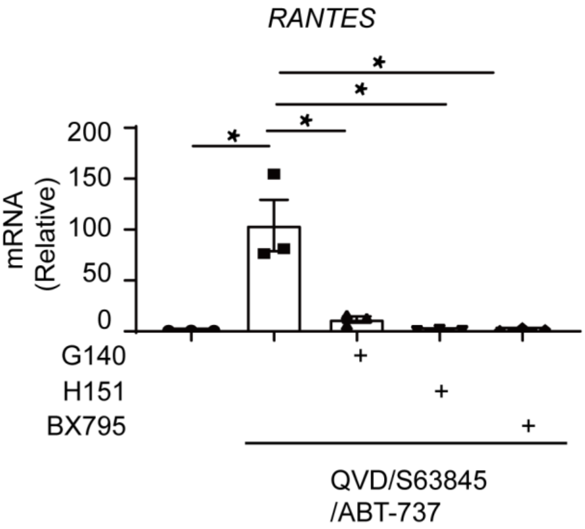

Figure S7. Mitochondrial apoptosis-induced IFN response depends on cGAS-STING pathway. Measurement of RANTES expression in BEAS-2B cells pretreated with 40 µM G140, 20 µM H151 or 10 µM BX795 for 1 h followed by additional treatment of 20 µM ABT-737, 20 µM S63845 and 20 µM QVD-OPh for 3 h. Mean expression of *RANTES* from three independent experiments was shown with standard errors for the mean. *$p < 0.05$ (Student's t-test),

Reference

1. Nakahira K, Kyung SY, Rogers AJ, Gazourian L, Youn S, Massaro AF, et al. Circulating mitochondrial DNA in patients in the ICU as a marker of mortality: derivation and validation. *PLoS Med*. **2013**, *10*, e1001577.
